# The Molecular Basis of Conformational Instability of the Ecdysone Receptor DNA Binding Domain Studied by *In Silico* and *In Vitro* Experiments

**DOI:** 10.1371/journal.pone.0086052

**Published:** 2014-01-23

**Authors:** Agnieszka Szamborska-Gbur, Grzegorz Rymarczyk, Marek Orłowski, Tomasz Kuzynowski, Michał Jakób, Agnieszka Dziedzic-Letka, Andrzej Górecki, Piotr Dobryszycki, Andrzej Ożyhar

**Affiliations:** 1 Department of Biochemistry, Faculty of Chemistry, Wrocław University of Technology, Wrocław, Poland; 2 Department of Physical Biochemistry, Faculty of Biochemistry, Biophysics and Biotechnology, Jagiellonian University, Kraków, Poland; Wake Forest University, United States of America

## Abstract

The heterodimer of the ecdysone receptor (EcR) and ultraspiracle (Usp), members of the nuclear receptors superfamily, regulates gene expression associated with molting and metamorphosis in insects. The DNA binding domains (DBDs) of the Usp and EcR play an important role in their DNA-dependent heterodimerization. Analysis of the crystal structure of the UspDBD/EcRDBD heterocomplex from *Drosophila melanogaster* on the *hsp27* gene response element, suggested an appreciable similarity between both DBDs. However, the chemical denaturation experiments showed a categorically lower stability for the EcRDBD in contrast to the UspDBD. The aim of our study was an elucidation of the molecular basis of this intriguing instability. Toward this end, we mapped the EcRDBD amino acid sequence positions which have an impact on the stability of the EcRDBD. The computational protein design and *in vitro* analyses of the EcRDBD mutants indicate that non-conserved residues within the α-helix 2, forming the EcRDBD hydrophobic core, represent a specific structural element that contributes to instability. In particular, the L58 appears to be a key residue which differentiates the hydrophobic cores of UspDBD and EcRDBD and is the main reason for the low stability of the EcRDBD. Our results might serve as a benchmark for further studies of the intricate nature of the EcR molecule.

## Introduction

The ultraspiracle (Usp) and ecdysone receptor (EcR) are members of the nuclear hormone receptor (NHR) superfamily [1]. They form the functional heterodimeric receptor for ecdysteroids which coordinates metamorphosis and major metabolic processes in insects [2]–[4]. The DNA-dependent dimerization of these two transcription factors takes place on a specific DNA fragment − the so-called hormone response element (HRE), and depends on their DNA-binding domains (UspDBD and EcRDBD, respectively) [5]. This process is crucial for modulation of expression of the target genes. Both the DBDs are necessary and sufficient to achieve specific binding to the target HRE [6]. The best characterized HRE for the EcR/Usp heterodimer is a quasi-palindromic element from the *hsp27* gene promoter (*hsp27_pal_*) [7], [8]. The DBDs are the most conserved domains of the nuclear receptors [9], [10]. Analysis of the crystal structure of the *Drosophila melanogaster* UspDBD/EcRDBD heterocomplex on the natural *hsp27_pal_* suggested an appreciable similarity between both domains [11]. Nevertheless, the chemical denaturation experiments and the circular dichroism (CD) spectra indicated an undeniably lower stability in solution and a lower α-helix content for the EcRDBD in comparison to the UspDBD. The EcRDBD deletion mutants, devoid of the C-terminal extension sequences (CTEs) also demonstrated instability, suggesting that this instability is an inherent property of the EcRDBD core [12]. The juxtaposition of the *D. melanogaster* EcRDBD instability and its structural similarity to the UspDBD has become a point of reference in understanding how the EcRDBD expresses its plasticity and adaptability described by Orłowski *et al.* [12].

The aim of our systematic research was an elucidation of the molecular basis of the remarkably low stability of the *D. melanogaster* EcRDBD molecule in comparison to the UspDBD. With this aim in view, we decided to identify the set of EcRDBD key amino acid residues which define this intriguing molecular property of the EcRDBD. To achieve this goal, computational methods were applied to a rational design of the EcRDBD mutants which proved to increase stability without losing the ability to interact specifically with the *hsp27_pal_* and UspDBD [5] with reference to the wild-type EcRDBD (EcRDBD_WT_). We performed *in silico* structure-based mutagenesis and mutant screening simulations together with a deep *in vitro* analysis of the EcRDBD conformational stability. We also compared the amino acid sequence and tertiary structure of the EcRDBD with the UspDBD and other nuclear receptor structures in order to indicate the specific set of amino acid residues which have an impact on the functionality and stability of the DBD. Our *in silico* and *in vitro* results identified several non-conserved amino acid residues within the α-helix 2 of the DBD hydrophobic core that are responsible for EcRDBD stability. Two crucial positions in this region (M49 and L58 amino acid residues) are apparently the key determinants of the low stability of the EcRDBD molecule in comparison to the UspDBD. The deep molecular analysis of the EcRDBD presented in our paper enhances overall knowledge of structural motifs and molecular mechanisms that have an impact on the stability and plasticity of the nuclear receptor DBD.

## Results

### Molecular modeling of the EcRDBD point mutant structures

The amino acid sequences and tertiary structures of the nuclear receptor DBDs are often analyzed in terms of similar scoring of their core regions (DBD fragments from C1 to C56 residues) and the CTEs including T- and A-box fragments [5], [6], [11]–[14]. The core regions are characterized by a high similarity level among nuclear receptor DBDs, whereas T- and A-box fragments show a wide diversity of amino acid sequences and secondary structure contents [11], [12], [15]–[18].

**Figure 1:**
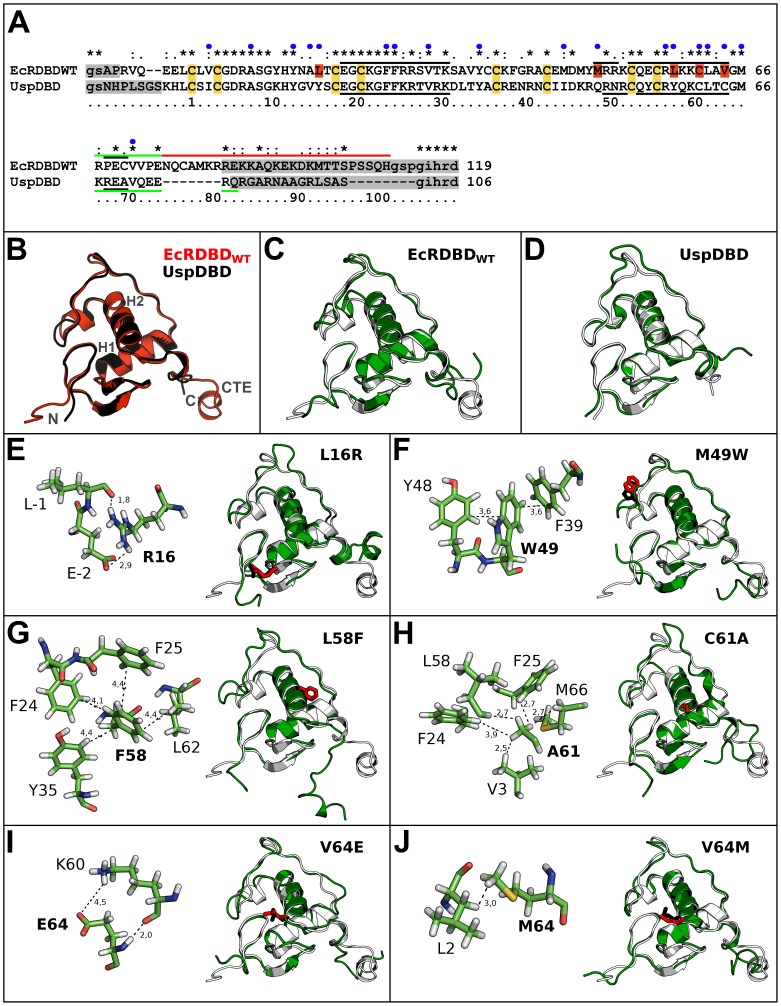
Analysis of amino acid sequences and 3D structures of the EcRDBD and UspDBD. (A) Alignment of EcRDBD_WT_ and UspDBD sequences. The alignment was done using the *needle* program (http://www.ebi.ac.uk/Tools/emboss/align/index.html) and revealed 46.0% of the sequences similarity and 38.1% of identity (*needle* score: 253.5). The residue numbering is relative to the first C residue coordinating the zinc ion of the DBD zinc module. The EcRDBD_WT_ sequence positions substituted by the RosettaDesign program [20], the conserved C residues coordinating the zinc ions and the terminal residues not visible in the crystal data [11] were highlighted in red, yellow and gray, respectively. Blue dots indicate residues that form the hydrophobic core that stabilizes the domain. The α-helix structures, T-box and A-box were marked outside of each sequence by black, green and red lines, respectively. (B) The crystal structures of the EcRDBD_WT_ (red) and UspDBD (black) are superimposed together by their Cα. The RMSD value for the superimposition of the DBD fragments (residues from C1 to M66 in both cases) is equal to 0.747 Å. The main α-helices 1 and 2 of the DBD core, N- and C-termini of the domains and the C-terminal extension were labeled by: H1 and H2, N, C and CTE labels, respectively. The domain structures were taken from the UspDBD/EcRDBD heterocomplex on a natural response element *hsp27_pal_* (PDB: 2HAN) [11]. (C) and (D) EcRDBD_WT_ and UspDBD energy-minimized structures (white) superimposed upon their structures obtained after a 10 ns time period of each MD simulation (green). (E–J, left) Side chain conformations of the chosen EcRDBD substituted residues together with their adjacent residues after 10 ns MD simulations. The shortest distances between the residues and salt bridges (in Å) are shown as dashed lines. (E–J, right) The whole EcRDBD point mutant energy-minimized structures (white) superimposed upon their structures obtained after a 10 ns time period of each MD simulation (green). The substituted residues were shown as sticks (before and after the MD simulations as black and red, respectively).

The EcRDBD and UspDBD from *D. melanogaster* are described by high amino acid sequences similarity and identity to each other. An alignment of their sequences was done with the *needle* program, using EMBOSS Pairwise Alignment Algorithms [19] and revealed sequences similarity of 46.0% and identity of 38.1% (*needle* score: 253.5) (see Figure 1A). The comparison of the EcRDBD and UspDBD crystal structures [11] was done by superimposition of Cα atoms (Figure 1B) and quantitatively described by a RMSD parameter. The degree of the structural similarity between the EcRDBD and UspDBD was described on the basis of the following fragment analysis: residues 1–56 (DBD cores), residues 1–66 (DBDs without the T and A boxes) and residues 1–74 (DBDs without the A-box). The RMSD values calculated for these fragments were respectively: 0.785 Å, 0.747 Å and 1.417 Å. The results indicated a high degree of structural similarity of both the DBDs, particularly between their extended cores (residues 1–66).

In order to map amino acid sequence positions that cause EcRDBD instability, we set about designing and producing the EcRDBD point mutants which prove increasing stability with reference to the EcRDBD_WT_. During the EcRDBD mutant structures design, i*n silico* structure-based mutagenesis and mutant screening simulations were performed using the RosettaDesign program [20]. RosettaDesign is suitable for protein design and has been used previously to stabilize protein structures [21]–[24]. The structure-based mutational analysis of the protein relies on searching for the lowest energy sequence for the template structure backbone. The crystal structure of the EcRDBD [11] was a structure template. All the EcRDBD residues directly interacting with the *hsp27_pal_*, the residues involved in the dimer interface of the UspDBD/EcRDBD complex [11], the eight cysteines coordinating the zinc ions and the A-box residues were held fixed. The remaining 31 residues of the 87-residue EcRDBD were allowed to be replaced by any amino acid in the redesign process. RosettaDesign evaluates the resultant protein sequences using an energy function [21], [25]–[28] and the mutant structures were created. During performing RosettaDesign runs, special attention was paid to the hydrophobic core residue substitutions that could play an important role in domain stability. To describe the contribution of the hydrophobic residues to domain stability, we defined the set of residues that form the EcRDBD hydrophobic core. The basis for determining that set of residues were: comparative studies of known nuclear receptor hydrophobic cores [9], [29]–[31], EcRDBD crystal structures analysis and the energy criterion established during the RosettaDesign calculation performed for the EcRDBD in non-substitution mode (for more details, see *Materials and Methods*). The EcRDBD hydrophobic core was established as follows: V3, A8, Y13, A15, L16, F24, F25, V29, Y35, M45, M49, R57, L58, C61, L62, V64, M66 and V71 residues. All of the nuclear receptor DBDs have a similar fold [9], [9,13]. Therefore, on the same principle, the corresponding UspDBD sequence positions were pointed out as forming the domain hydrophobic core. The set of amino acid residues was defined respectively as: I3, A8, Y13, V15, Y16, F24, F25, V29, Y35, I45, Q49, R57, Y58, C61, L62, C64, M66 and V71 residues.

The EcRDBD mutants' design was carried out in seven rounds with different substitution parameters and tens of various outputs of the multiple mutant sequences and structures were obtained (data not shown). All the mutant sequences and structures were evaluated by the RosettaDesign energy function and twenty five of the best scored mutants were taken into further consideration. Simultaneously, a thorough examination of the best mutations suggested by the program was done on the basis of: i) general knowledge about the structural motifs that increase proteins' stability [32]–[39], ii) a visual inspection of the designed mutant structures and iii) a comparison of the resultant sequences with other nuclear receptor DBD sequences (see Figure S1 for DBD sequence alignment). This comprehensive approach led us to extract six individual substitutions designated by RosettaDesign (L16R, M49W, L58F, C61A, V64E and V64M) (see Supporting Information S1 for the EcRDBD mutant structures). They have structural justification and apparently form new intramolecular interactions and thereby could improve EcRDBD stability (Figure 1E–J). The contributions of the six substitutions in EcRDBD stability were evaluated by the RosettaDesign scoring function [21], [25], [27], [28]. The point mutant structures' scores ordered from best to worst were: V64E>V64M>C61A>L16R>M49W>L58F.

Following suggestions given by RosettaDesign, the L16R substitution caused an exchange from the hydrophobic residue with the hydrophilic one, near the N-termini end of the domain (Figure 1E). It contributes to the creation of a strong salt bridge between the guanidinium group of R16 residue and carbonyl oxygen atom of L(−1) residue and stiffens the N-termini end and holds it closer to the domain core. Additionally, the alignment of many of the known nuclear receptor amino acid sequences yielded the selection of two DBDs having R residue at the corresponding position: the nuclear hormone receptor HR38 from a fruit fly (*d*HR38) and the nerve growth factor IB-like receptor from a rat (NGFI-B) (UniProt identifiers: P49869 and P22829, respectively) (see Figure S1). It gave us an additional clue that the L16R substitution could be important in our approach.

An indole group of W49 residue is located between two aromatic rings of F39 and Y48 residues (Figure 1F). The three aromatic rings are almost perpendicular to one another, and the dihedral angles between them were 94.4° and 80.3° for the F39-W49 and W49-Y48 pairs, respectively. They seem to create tough edge-to-face interactions [40] between their aromatic chromophores located in a small cavity, close to the domain surface.

The replacement of L58 residue by F residue significantly increases the aromatic interactions in the middle of the EcRDBD hydrophobic core (Figure 1G). This substitution was especially interesting because of the Y residue at the corresponding sequence position in the UspDBD (see Figure 1A). The result of this molecular modeling called our attention to one of the key differences between the hydrophobic cores of the UspDBD and EcRDBD_WT_ (see Figure 1A) which could be the main reason for the completely different stability levels of both domains. Moreover, an F residue is found at the respective sequence position in the mentioned *d*HR38 and NGFI-B DBDs, as well as in the human thyroid hormone receptors alpha and beta (TRα and TRβ, respectively) and the peroxisome proliferator-activated receptor alpha (PPARα), (UniProt identifiers: P49869, P22829, P10827, P10828 and Q07869, respectively) (see Figure S1).

Three of the six chosen substitutions: C61 to A residue and V64 to E or M residues, obtained lower RosettaDesign score values than the rest of the mutations. The A61 residue keeps hydrophobic contacts with V3, F24, F25, L58 and M66 residues, situated in the DBD core (Figure 1H). Removal of the sulfhydryl group of C61 residue may prevent ionization within this environment. Nevertheless, the crucial C61 residue, which stabilizes the equilibrium structure of the DBD fold, is highly conserved from among the nuclear receptor DBDs [9], [29], [41]. As described by Low *et al.* (2002), replacing the C61 residue in the DBD core by an A residue destabilized the DBDs of both the estrogen and glucocorticoid receptors (ERDBD and GRDBD, respectively) [41]. On the other hand, the C61A substitution was described as improving the DBD stability in the case of the retinoid X receptor (RXRDBD) [42]. Although mutation C61A seemed to be a risky solution to the issue of EcRDBD instability, we decided to check how one of the best scored substitutions would influence the DBD obtained from the insect NHR.

The V64E mutation replaces the hydrophobic residue with the hydrophilic one and could be beneficial with respect to its localization on the EcRDBD surface (Figure 1I). The E64 residue enables us to create a salt bridge with the R60 residue. Conducting the analysis of all the RosettaDesign runs, we noted that the V64E mutation is repeated by the program. These observations confirm one of the described features of RosettaDesign: the specific residue pair contacts that describe the electrostatic interactions and disulfide bonds within the protein structure are favored in the design procedure [21], [43].

Finally, the V64M substitution also changes the non-polar character of the V residue to the more polar M residue at the domain surface (Figure 1J). The substitution V64M was especially interesting with regard to other NHRs that have methionine at the exact corresponding positions of their DBD amino acid sequences. These are: Usp from honeybee (*Apis mellifera*), Uruçu bee (*Melipona scutellaris*), Colorado potato beetle (*Leptinotarsa decemlineata*), human retinoid X receptor alpha (RXRα) and human farnesoid X-activated receptor beta (FXRβ) (UniProt identifiers: Q9NG48, Q5MBF7, Q4W6C8, P19793 and Q96RI1, respectively) (see Figure S1).

Additionally, the selected amino acid positions (16, 49, 58, 61 and 64) of the EcRDBD sequence were analyzed in *D. melanogaster* and other species (Figure S1). The analysis showed that L16, M49, L58, and V64 residues are strongly conserved across EcRDBDs in disparate species. As mentioned above some of the L, M, L and V residues were also found at the corresponding sequence positions in several nuclear receptors. However, this set of residues (16, 49, 58 and 64, respectively) is not present in the UspDBD sequences and appears to be a watermark of EcRDBDs. According to the analysis and literature, C61 residue is fully conserved from among the nuclear receptor DBDs [9], [29], [41].

### Molecular dynamics simulations of the EcRDBD mutants

An independent study of the influence of the six substitutions on conformation and flexibility of the whole EcRDBD molecule or its particular regions was carried out. The mutant structures, each containing one of the six mutations, were analyzed using molecular dynamics (MD) simulation techniques. The MD simulations provide general information about molecular mobility in time on an atomic level. The initial coordinates of the structures were defined after energy minimization. Deviations (measured in Å) from the mean position of the MD simulation trajectories for Cα atoms were determined using the RMSD parameter. Each DBD structure obtained after 10 ns of the MD simulation was averaged in the last 300 ps of its trajectory using the *ptraj* program [44] (see Supporting Information S1 for averaged structure of DBDs). All of these structures are presented in Figure 1C–J (green structures). The analysis of conformational changes of each individual EcRDBD_WT_ and UspDBD (panels C and D of Figure 1, respectively) with reference to their initial structures (Figure 1C–D, grey structures), yields insight into differences in the domains' behavior independently of the crystal restrains. We wanted to investigate, if removal of the DNA response element and the UspDBD partner produce perceptible conformational changes in the simulated EcRDBD_WT_ data set. Moreover, the analysis of the MD simulation trajectories of the EcRDBD point mutants could facilitate providing general characteristics of the EcRDBD structure. In some cases, the effects of mutation of the nuclear receptor DBDs could be meaningful. The substitutions would possibly mimic an allosteric effect of the DNA or/and DBD partner as described for the GRDBD [45].

As is shown in Figure 1 (panels C–J), the mutual orientation of the two main α-helices (the so-called α-helices 1 and 2) forming the backbones of the DBDs did not change after 10 ns of the MD simulation. In all cases, the α-helices 1 and 2 lie antiparallel to each other, almost identical to before the MD simulations. Drastic conformational changes were observed for both the N-terminal ends and the CTE sequences of the wild-type and mutated EcRDBDs. This is clearly noticeable in the case of the L58F mutant (Figure 1G). The comparison of the UspDBD structure before and after the MD simulation showed slight differences between both backbone conformations (Figure 1D). Importantly, this 78-residue domain has a very short CTE sequence. In the crystallographic data, the UspDBD can be seen with shorter N- and C-terminal ends, shorter by 9 amino acids than the EcRDBD_WT_ in total (see Figure 1A) [11]. Therefore, predominantly only the UspDBD core can be analyzed by the MD methods.

**Figure 2:**
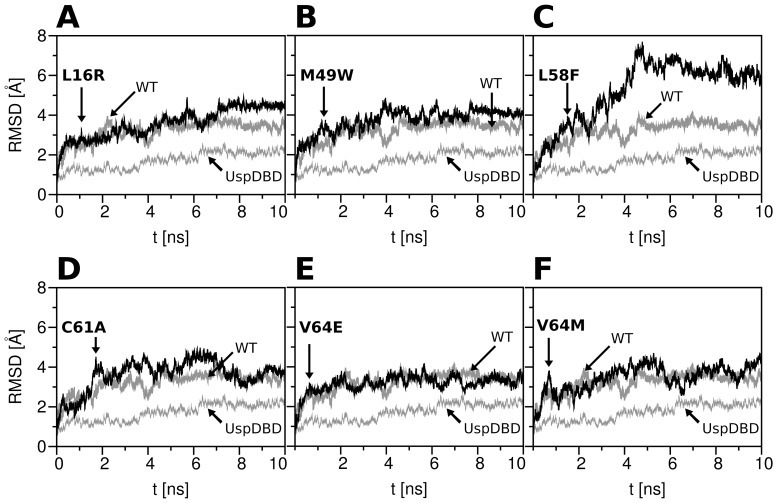
Root-mean-square deviation (RMSD) profiles with respect to the EcRDBD mutant structures during MD simulations. The trajectories of the backbone root-mean-square deviation (RMSD) of the EcRDBD point mutant structures (black lines) in comparison with the EcRDBD_WT_ (gray lines, WT) and the UspDBD (gray, thin lines). The RMSDs were calculated for 10 ns MD simulations at 300 K with respect to energy-minimized structures. The thermalisation time up to 300 K is not shown.

A detailed comparison between conformational changes of the point mutant backbones and data collected for the EcRDBD_WT_ and UspDBD was shown in Figure 2. The largest variations of RMSD values were noted for the point mutant structures containing substitutions within their hydrophobic cores, such as L58F and C61A (panels C and D of Figure 2, respectively). The EcRDBD structures mutated near or at their surfaces (the L16R, M49W, V64E and V64M point mutants analyzed in Figure 2, panels A, B, E and F, respectively) were characterized by comparable RMSD changes as those of the EcRDBD_WT_. Monitoring of the L58F mutant structure trajectory suggested significant motions of its backbone (Figure 2C). This is probably due to the replacement of the smaller L residue with the bigger F residue in the middle of the hydrophobic core, and consequently the impact of the F58 residue on the F24, F25, Y35 and L62 residues (compare Figure 2C with Figure 1G). It is clearly noticeable, that the MD simulation system, containing the L58F mutant structure, was the only one which required a longer simulation time to rearrange the domain hydrophobic core and to achieve an equilibrium state. The parallel calculations of RMSD changes during the MD simulations were also done for extended cores of the mutated and wild-type EcRDBDs (the same residue range for all structures: from C1 to M66). The shapes of the RMSD profiles were similar to those calculated for the full-length domains, but the final RMSD values were always much lower (data not shown). The RMSD profile of the UspDBD backbone differs from the RMSD profiles calculated for both the EcRDBD_WT_ and point mutants (Figure 2). There were no such significant folding changes as noticed for both the wild-type and mutated EcRDBDs. Finally, all the simulated DBD structures were characterized with low backbone fluctuations at least from the 8-th nanosecond of each simulation.

**Figure 3:**
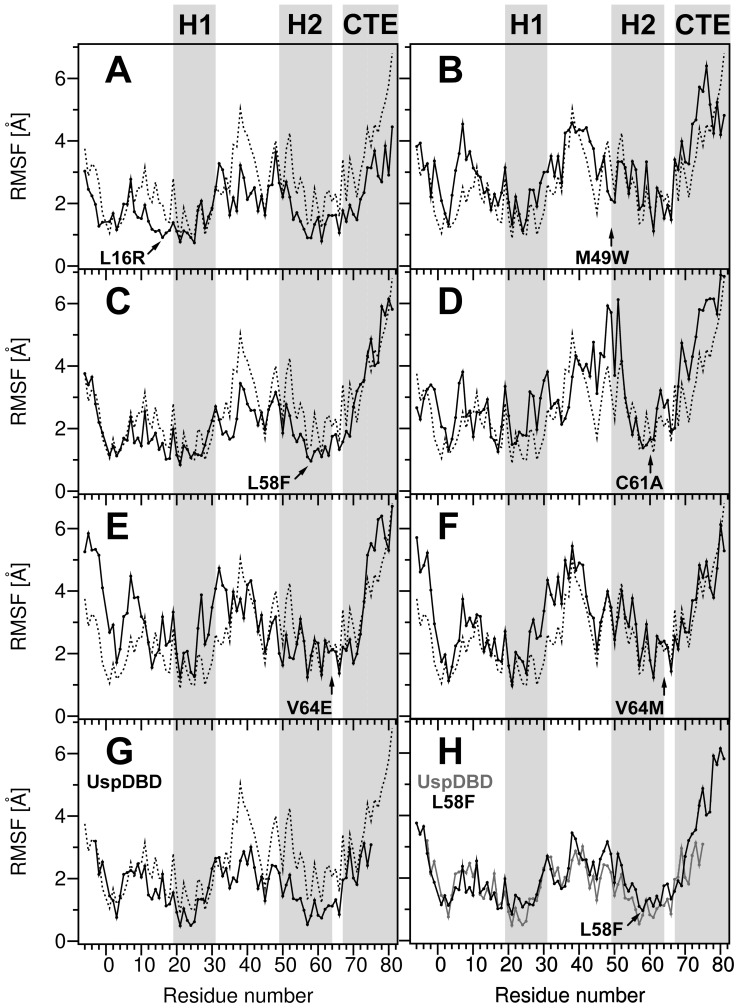
Root-mean-square fluctuation (RMSF) profiles with respect to EcRDBD mutant structures during MD simulations. The side chain root-mean-square fluctuations (RMSF) of the EcRDBD point mutants and the UspDBD (solid lines) in comparison with the EcRDBD_WT_ (dashed lines) calculated for the last 2 ns time period of MD simulations (from 8^th^ to 10^th^ nanosecond), panels A–G. Panel H represents the comparison between the RMSF profiles of the L58F point mutant (black line) and the UspDBD (gray line). The substituted EcRDBD positions are displayed by arrows. The secondary structure elements (α-helices 1 and 2) and C-terminal extension (CTE) sequence [5], [6], [12] are indicated by gray areas and labeled by: H1, H2 and CTE labels.

The amplitude of the side chain motions of all the DBDs was investigated for the time frame of 8–10 ns of each MD simulation and is presented by RMSF parameter profiles in Figure 3. A characteristic feature of all the RMSF profiles is the slight motion range of residues forming the α-helices 1 (H1) and 2 (H2), together with a high level of the N and C-terminal ends fluctuations. It can be especially seen for the wild-type and mutated EcRDBDs. Interestingly, RMSF values are lower within the α-helix 1 than the α-helix 2 of the EcRDBD_WT_. According to the presented results, the CTE sequence is the most labile fragment of each domain. This concurs with previously published results showing that, in contrast to the vertebrate nuclear receptors, the EcRDBD CTE sequence could be involved in DBD core stabilization [12]. The most significant side chains' variation of the DBD cores are observed for the M49W, C61A, V64E and V64M point mutant structures (panels B, D, E and F of Figure 3, respectively), as opposed to the L16R and L58F point mutant structures (panels A and C of Figure 3, respectively), proving a lesser degree of change of core fluctuations (about 1 Å). A particularly interesting RMSF profile was obtained for the L58F point mutant structure. This DBD model was characterized by the greatest conformational changes of its Cα atoms in 1–5 ns of the MD simulation time frame (see Figure 2C). Finally, its side chains were fluctuating less than the EcRDBD_WT_ side chains (Figure 3C) during the last 2 ns of the MD simulations. According to our expectations, the UspDBD side chains displayed low fluctuating movements (from 0.5 to 3.2 Å of RMSF, see Figure 3G), which correlates well with the UspDBD stability previously observed in the *in vitro* experiments [12].

Interestingly, both RMSF profiles of the L58F point mutant and UspDBD turned out to be similar to each other (Figure 3H). The comparison of side chains fluctuation ranges performed for the L58F point mutant and UspDBD structures showed that in both cases the RMSF values stayed on the same level. Even though slight differences in RMSF values (±0.2 Å) are seen within H1 and H2 regions of both domains, they are negligible (Figure 3H). The substitution of L58F seems to make the EcRDBD resemble the UspDBD. According to our computational analysis the L58F mutation is particularly worth exploring because of the Y residue at the corresponding sequence position in the UspDBD (see Figure 1A). The aromatic Y and F residues at position 58 of the DBD core probably have similar influence on the domain structure.

### Determination of involvement of the designed substitutions on EcRDBD functionality and its secondary structure content

To validate the computational modeling results, a series of EcRDBD constructs that coded respective EcRDBD mutants were generated using the site-directed mutagenesis [46]. Next, the wild-type and point mutated EcRDBDs and the UspDBD were overexpressed in *E. coli* and then were purified to homogeneity (data not shown).

**Figure 4:**
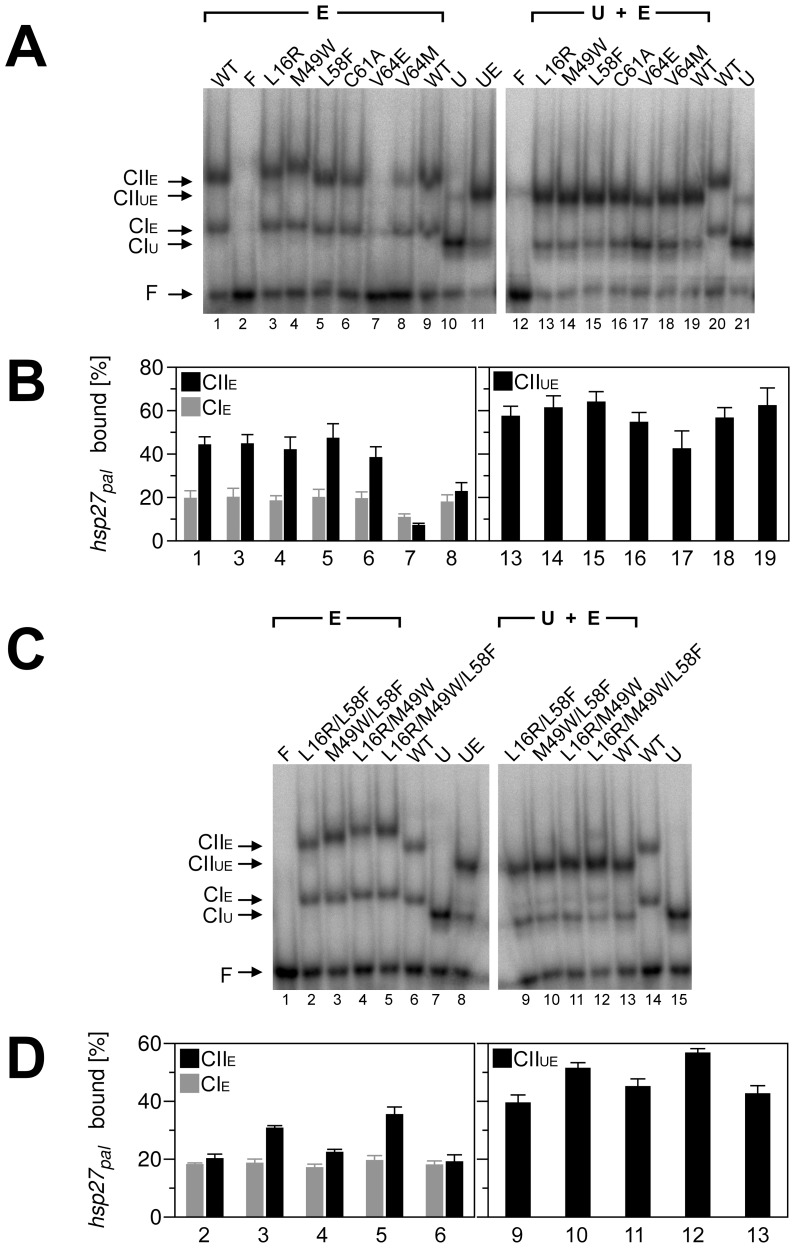
The analysis of the EcRDBD mutants' binding to the *hsp27_pal_*. The electrophoretic mobility-shift assays (EMSA) were conducted with the indicated EcRDBD (E) or the UspDBD (U), separately (panel A, lanes 1, 3–10 and 20–21; panel C, lanes 2–7 and 14–15) or with an equimolar mixture of both the indicated EcRDBD and UspDBD (panel A, lanes 11, 13–19; panel C, lanes 8–13). The protein (CI – monomer, CII – dimer) complexes formed with the *hsp27_pal_* were denoted as: U, E or UE for the UspDBD, indicated EcRDBD and both DBDs heterodimer, respectively. F, free DNA probe, WT – EcRDBD_WT_. The WT, U, UE and F lanes were included as controls. The positions of the corresponding complexes are marked on the left. The total protein concentrations were: 200 nM (panel A, lanes 1, 3–8 and 13–19) and 50 nM (panel C, lanes 2–6 and 9–13), using half of the amounts of each component that were used with a single DBD. The EMSA results were quantitatively analyzed as described previously [12], which is shown in panel B (data from panel A, lanes 1, 3–8 and 13–19, respectively) and panel D (data from panel C, lanes 2–6 and 9–13, respectively).

First of all, we wanted to verify if the selected EcRDBD residues (L16, M49, L58, C61 and V64) do not affect protein-protein and protein-DNA interactions in the UspDBD/EcRDBD-*hsp27_pal_* complex [11]. The influence of the designed substitutions (L16R, M49W, L58F, C61A, V64E and V64M) on the binding abilities of the mutated DBDs to both the *hsp27_pal_* response element and the UspDBD was determined by the electrophoretic mobility shift assay (EMSA) experiments [47]. The effects caused by each of the mutations on the previously observed DNA-dependent homo- and heterodimerization and the quantitative and qualitative analyses of these processes are illustrated in Figure 4A–B. According to the results of the EMSA experiments published by Niedziela-Majka *et al.* [5], the *D. melanogaster* EcRDBD_WT_ and *hsp27_pal_* create two types of complexes, the homodimer (Figure 4A; lane 1 and Figure 4B; bar 1) and the heterodimer formed with the UspDBD (Figure 4A; lane 19 and Figure 4B; bar 19). Moreover, the EcRDBD_WT_/UspDBD heterocomplex affinity to the *hsp27_pal_* is higher than noticed for the EcRDBD_WT_ homodimer [5], [12], [48]. Here, the EMSA experiments showed a significant influence on the homo- and heterodimers' specific interactions with the *hsp27_pal_* for two of the analyzed substitutions. Binding the *hsp27_pal_* by the EcRDBD homodimers was reduced significantly by the V64M substitution (Figure 4A; lane 8 and Figure 4B; bar 8), whereas, the V64E point mutant demonstrated a noticeable DNA-binding defect (Figure 4A; lane 7 and Figure 4B; bar 7). The heterocomplex of the UspDBD and the V64E point mutant demonstrated a decreased DNA-binding affinity to the *hsp27_pal_* too (Figure 4A; lane 17 and Figure 4B; bar 17). This clearly indicates that the substitutions at position 64 have a destructive influence on the EcRDBD structure and in consequence on the *hsp27_pal_*-binding affinity of the EcRDBD. The homo- and heterodimerization ability of the C61A point mutant in the presence of the DNA was moderately decreased (Figure 4A–B; lanes 6 and 16, bars 6 and 16). Interestingly, the heterodimerization runs with moderate difference in efficiency for the rest of the analyzed point mutants as well as for the EcRDBD_WT_. In spite of the M49W mutation, the EcRDBD retained its ability to bind specifically to both the *hsp27_pal_* and UspDBD (Figure 4A; lane 14 and Figure 4B; bar 14). The M49W substitution increased the hydrophobic character of the domain and probably strengthened the aromatic interactions between Y48 and F39 residues (see Figure 1F). This stiffened part of the DBD structure is near the amino acid residues involved in forming the dimer interface (M47, Y48 and R51 residues) [11]. Therefore, the M49W substitution could change the EcRDBD binding affinity to the *hsp27_pal_*. Nevertheless, the homo- and heterodimerization levels of the M49W point mutant (Figure 4A–B; lanes 4 and 14, bars 4 and 14 for homo- and heterodimers, respectively) were similar to the EcRDBD_WT_ (Figure 4A–B; lanes 1 and 19, bars 1 and 19). Similar results were obtained for the L16R point mutant (Figure 4A–B; lanes 3 and 13, bars 3 and 13). Finally, the L58F point mutant was characterized by the highest DNA-binding affinity as both the homo- and heterodimers (Figure 4A–B; lanes 5 and 15, bars 5 and 15). The F58 residue in the middle of the EcRDBD hydrophobic core has an impact on the F24, F25, Y35 and L62 residues (see Figure 1G). Consequently, the rearrangement of the domain hydrophobic core including the H1 and H2 α-helixes (see Figure 2C and 3C) has an influence on its ability to form specific binding of both the *hsp27_pal_* and UspDBD.

**Figure 5:**
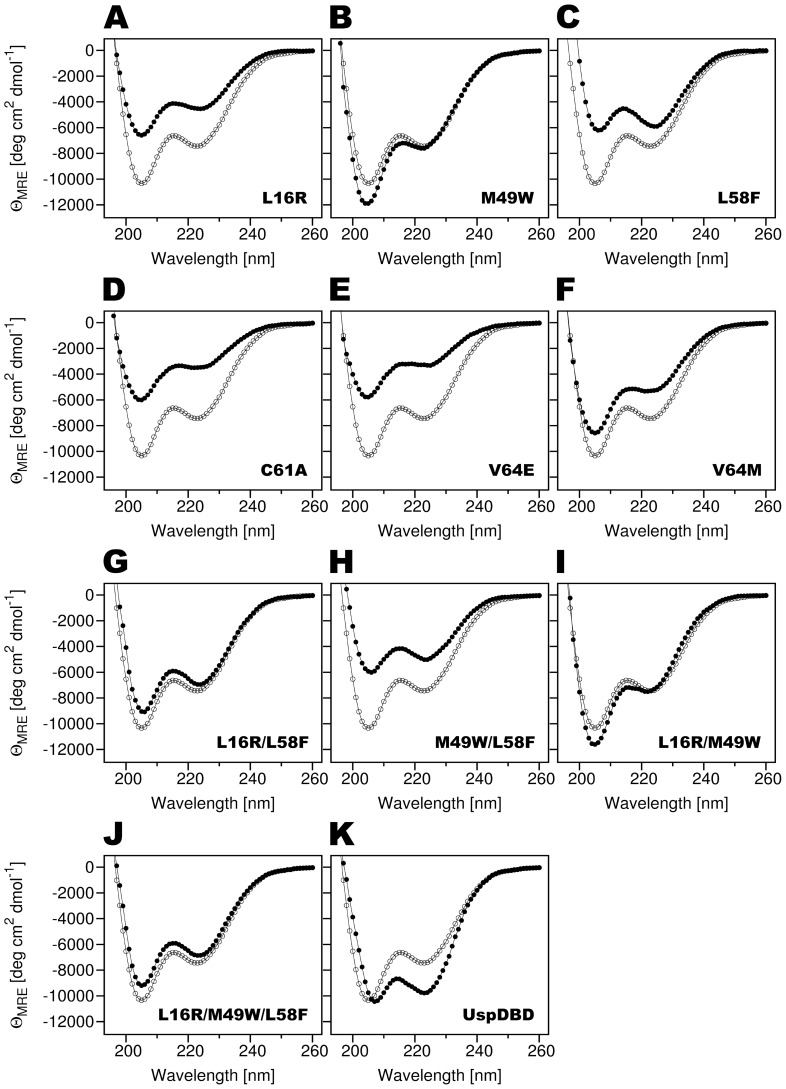
The far-UV CD spectra of the EcRDBD mutants. The CD spectra were recorded at 20°C, at 0.15 mg/ml protein concentrations, with a path-length of 0.1 cm. Three scans (speed 20 nm/min., response time 4 sec., sensitivity standard 100 mdeg) were averaged to one smooth spectrum. For each spectrum, the medium base line has been subtracted and the mean residue ellipticity (Θ_MRE_ in degree×centimeter^2^×decimole^−1^) versus wavelength is shown. Compare each of the EcRDBD mutants and UspDBD (filled circles) with the EcRDBD_WT_ (open circles).

To characterize the influence of the L16, M49, L58, C61 and V64 residues' substitution on the EcRDBD structure, the CD spectra for all the mutated domains were recorded (Figure 5). A quantitative estimation of the secondary structure content was calculated using the CDPro software package [49] and is summarized in Table 1. Each of the designed EcRDBD substitutions has different effects on the secondary structures of the domain. The C61A and V64E point mutants were described by significantly different CD spectra than the EcRDBD_WT_ (panels D and E of Figure 5). These differences are mainly a consequence of losing α-helical structures for the benefit of β-strands (Table 1). The characteristics of the C61A and V64E point mutants structures concur with the EMSA results. As described above, the V64E point mutant is characterized by a severe reduction of its affinity to the *hsp27_pal_* as homodimer and moderately as heterodimer (see Figure 4A–B; lanes 7 and 17, bars 7 and 17). The C61A substitution has a moderate influence on the binding affinity towards the *hsp27_pal_* as homo- and heterodimer (Figure 4A–B; lanes 6 and 16, bars 6 and 16). Surprisingly, the V64E point mutant achieved the highest score in computational analysis performed using the RosettaDesign program (see Table 2). The L16R, L58F and V64M, point mutants are moderately different from one another in their secondary structures (Figure 5, panels A, C and F, respectively). A reduction in α-helix content for the benefit of β-strands was noticed in this group of the EcRDBD point mutants (Table 1). However, the EMSA experiments showed these changes are tolerated by the EcRDBD and do not lead to significant DNA-binding defects, at least in the case of the L16R, L58F homo- and heterodimers (Figure 4A–B; lanes 3, 5, 13 and 15, bars 3, 5, 13 and 15). A general resemblance between the CD spectra of the M49W point mutant and the EcRDBD_WT_ has been noticed (Figure 5B). The content of their secondary structure elements is also similar (Table 1).

**Table 1:** Quantitative estimation of the secondary structure contents for the EcRDBD_WT_, EcRDBD mutants and UspDBD.

Protein	α-helix [%]			β-strand [%]			Turns [%]	Unordered[%]	RMSD
	Regular	Distorted	Total	Regular	Distorted	Total			
**EcRDBD**									
WT	12.4±0.4	10.7±0.5	23.1±0.9	6.9±1.2	5.5±0.8	12.4±2.0	14.1±1.3	50.4±2.6	0.059
L16R	7.0±0.9	8.3±0.8	15.3±1.7	14.8±0.9	7.9±0.4	22.7±1.3	14.5±1.3	47.5±2.6	0.019
M49W	11.5±0.7	10.8±0.2	22.3±0.9	6.1±0.9	5.0±0.3	11.1±1.2	14.0±0.7	52.6±1.5	0.048
L58F	9.1±0.8	9.5±0.9	18.6±1.7	13.9±1.5	8.4±1.5	22.3±3.0	17.1±1.4	42.0±3.2	0.091
C61A	3.1±0.7	3.3±0.5	6.4±1.2	16.1±2.5	10.2±1.4	26.3±3.9	14.2±1.3	53.1±4.1	0.032
V64E	2.6±0.6	4.8±0.4	7.4±1.0	18.3±2.4	8.1±1.3	26.4±3.7	14.2±1.2	52.0±4.0	0.032
V64M	8.5±0.6	9.0±0.9	17.5±1.5	11.9±2.1	7.3±1.1	19.2±3.2	15.2±1.8	48.1±4.0	0.025
L16R/L58F	12.5±0.2	11.2±0.4	23.7±0.6	9.2±2.6	6.5±1.6	15.7±4.2	15.4±0.9	45.2±3.3	0.054
M49W/L58F	8.1±0.4	8.3±0.5	16.4±0.9	15.4±2.0	8.8±0.8	24.2±2.8	16.3±0.8	43.1±3.4	0.040
L16R/M49W	12.9±0.5	11.8±0.5	24.7±1.0	5.2±1.6	4.8±0.2	10.0±1.8	12.9±1.3	52.4±1.4	0.038
L16R/M49W/L58F	11.2±0.4	10.6±0.4	21.8±0.8	9.1±2.7	6.2±1.4	15.3±4.1	14.8±1.4	48.1±3.3	0.041
**UspDBD**	14.8±0.6	13.4±0.6	28.2±1.2	6.5±0.4	5.7±0.2	12.2±0.6	16.2±1.3	43.4±1.6	0.044

Calculation were carried out for the 119-amino acid EcRDBD polypeptide and for the 106-amino acid UspDBD polypeptide spectra at 20°C. The SELCON3, CDSSTR and CONTIN/LL programs were used and results were averaged. RMSD is a CONTIN/LL fit parameter, with low values indicative of close correspondence between calculated secondary structure and experimental data [23].

**Table 2:** Guanidine hydrochloride (GdmCl) half concentration (C_1/2_) and melting temperature (T_m_) obtained for the EcRDBD_WT_, its mutants and the UspDBD in chemical and thermal denaturation experiments in comparison with the RosettaDesign scoring.

Protein	GdmCl C_1/2_ [M]	T_m_ [°C]	RosettaDesign *score*
UspDBD	1.94±0.11	58.13±2.01	
EcRDBD			
WT	0.74±0.10	51.17±2.54	−32.40 (−41.57)^*^
L16R	0.80±0.15	45.11±3.47	−42.82
M49W	1.43±0.11	44.33±2.12	−42.50
L58F	1.00±0.07	55.64±2.63	−42.18
C61A	0.23±0.04	31.57±4.15	−43.57
V64E	0.33±0.08	36.66±2.22	−45.05
V64M	0.34±0.09	39.98±1.50	−44.79
L16R/L58F	0.44±0.13	53.75±3.43	−43.92
M49W/L58F	1.31±0.11	54.22±1.06	−43.47
L16R/M49W	0.57±0.08	44.13±1.66	−44.92
L16R/M49W/L58F	0.73±0.12	44.85±2.91	−44.72

**Figure 6:**
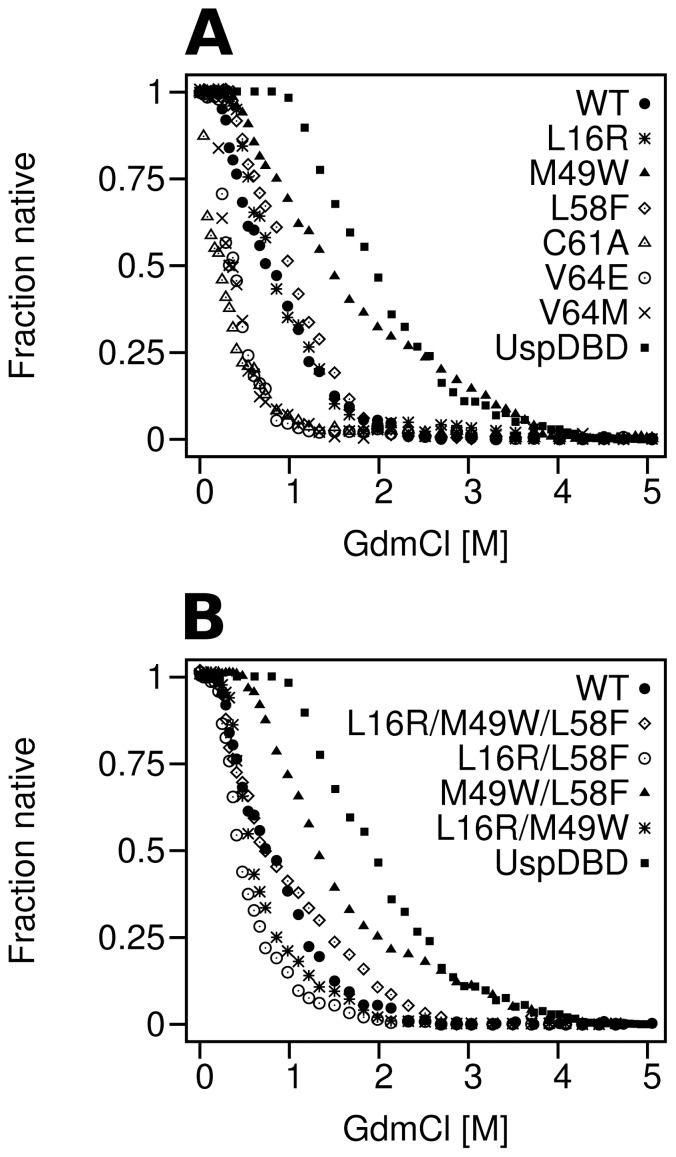
The GdmCl denaturation profiles of the EcRDBD mutants. The chemical denaturation experiments were conducted at 20°C, at protein concentrations of 2.5 µM, and respective denaturation curves were recorded using fluorescence measurement. Further details are given under *Materials and Methods*. One of the three representative profiles for this experiment is shown. Panel A: filled circles – EcRDBD_WT_ (WT), asterisks – L16R, filled triangles – M49W, open diamonds – L58F, open triangles – C61A, open circles – V64E, x symbols – V64M, filled squares – UspDBD. Panel B: filled circles – EcRDBD_WT_ (WT), open diamonds – L16R/M49W/L58F, open circles – L16R/L58F, filled triangles – M49W/L58F, asterisks – L16R/M49W, filled squares – UspDBD.

**Figure 7:**
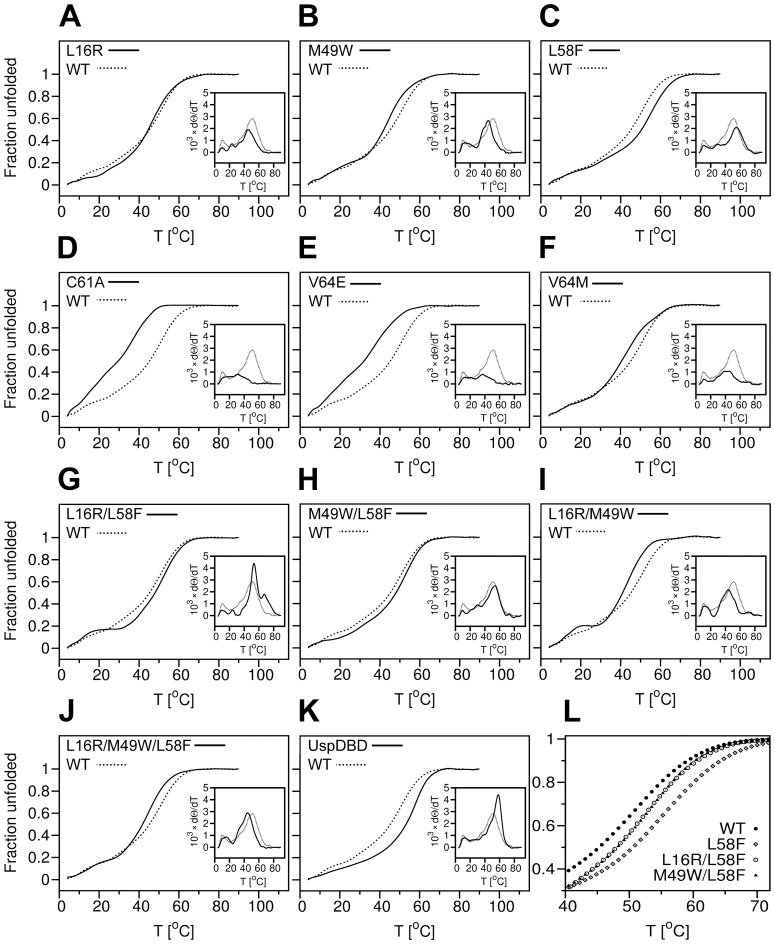
The thermal denaturation profiles of the EcRDBD mutants. The normalized denaturation curves of the temperature-induced unfolding of the EcRDBD mutants and UspDBD (solid lines in both cases) are shown in comparison with the EcRDBD_WT_ (dashed lines, WT). The thermal denaturation curves were obtained by monitoring changes of ellipticity at 222 nm, with a probe heating speed of 1°C/min. and a time-interval measurement of 20 seconds at protein concentrations of 0.15 mg/ml. The following ellipticity measurements which are shown were carried out independently four times and averaged to one curve each. Insets – The first derivatives of the CD unfolding curves (dΘ/dT) of the EcRDBD mutants and UspDBD (solid lines in both cases) were compared with the EcRDBD_WT_ data (dashed lines, WT). The dΘ/dT curves were calculated using Jasco Analysis software (JASCO Corporation, Japan) and the apparent melting temperature (T_m_) of each denaturation process was determined (see Table 2). Panel L – The CD unfolding profiles of the three EcRDBD mutants can be characterized by increased thermostability (L58F – open diamonds, L16R/L58F – open circles and M49W/L58F – filled triangles) in comparison with the EcRDBD_WT_ (filled circles, WT). The comparison of the denaturation profiles was done in the temperature range from 40°C to 70°C.

**Figure 8:**
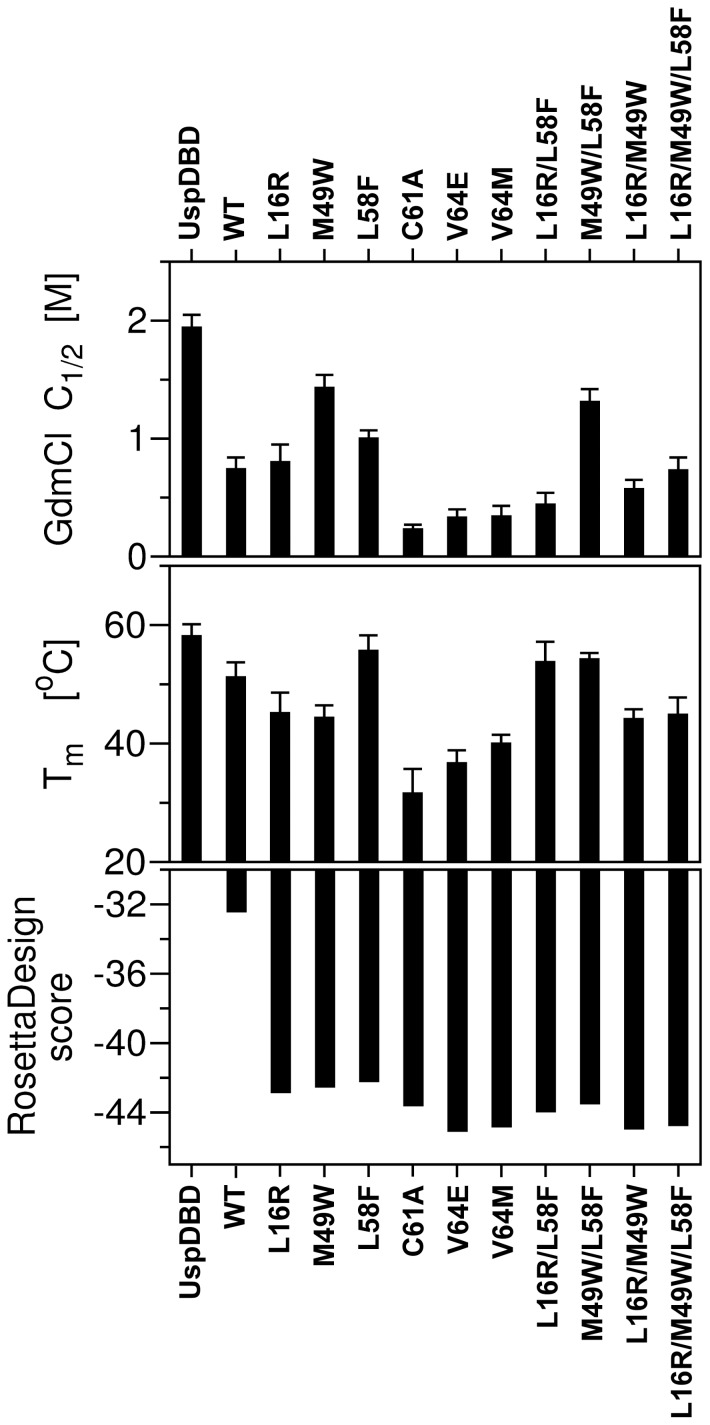
Graphical presentation of guanidine hydrochloride (GdmCl) half concentration (C_1/2_), melting temperature (T_m_) and the RosettaDesign scoring obtained for the EcRDBD mutants in comparison with the EcRDBD_WT_ and UspDBD. Aligned histograms were shown for convenience of the data comparative analysis.

The GdmCl concentration was taken for 50% of the protein fraction unfolded each and melting temperature values were assigned to the thermal denaturation curve derivative maxima (see Figure 6, Figure 7, insets and Figure 8). An asterisk sign for the EcRDBD_WT_ score indicates value calculated for a side chain rotamers redesign structure (without any substitutions). The lower RosettaDesign score value the better structure [17].

The analysis of the EMSA results and CD spectra showed that substitution of only one position in the EcRDBD amino acid sequence (i.e. V64 residue) changed the secondary structure content of the EcRDBD and caused a significant DNA-binding defect. Mutation of other analyzed EcRDBD residues (L16, M49, L58 and C61) turned out to have various impacts on the DBD structure. Even though some of the substitutions had significant influence on the EcRDBD structure, i.e. L16R and L58F, they were structurally adopted due to the high plasticity of the domain. Therefore, differences in the CD spectra of the mutated EcRDBD did not necessarily align with differences in DNA-binding affinity.

### An experimental stability evaluation of EcRDBD point mutants

To determine how the point mutations affected the stability of the EcRDBD, chemical denaturation experiments were carried out. The unfolding curves were obtained by monitoring intrinsic fluorescence (Figure 6A). We quantified the chemical stability of the domains by taking an apparent GdmCl concentration (called as C_1/2_; GdmCl concentration in which half of the protein fraction is denatured) from the unfolding curves (see Table 2). Interestingly, EcRDBD stability was increased by the M49W substitution to the largest extent (C_1/2_ 1.43 M) in comparison to the EcRDBD_WT_ (C_1/2_ 0.74 M). The substitutions of L16 and L58 residues slightly stabilized the domain (C_1/2_ equals to 0.80 M and 1.00 M, respectively). The denaturation profile of the L16R point mutant largely coincides with the EcRDBD_WT_ profile (Figure 6A, asterisks and filled circles, respectively). Notably, the C61A, V64E and V64M substitutions not only failed to improve EcRDBD stability but they even reduced it (Figure 6A, open triangles, open circles and x symbols, respectively). The C61A and V64E substitutions induced major changes in the secondary structure of the EcRDBD (see Figure 5, panels D and E and see Table 1). By contrast, the V64M point mutant was characterized by moderate changes in its CD spectrum in comparison to the EcRDBD_WT_ (see Figure 5F). These results were largely inconsistent with the RosettaDesign scoring. Although during the computational design, the C61A, V64E and V64M point mutants were distinguished with the best scores (Table 2), they were experimentally proven to be the most unstable domains. On the other hand, the structures of the L16R, M49W and L58F point mutants, which appeared to be more stable than EcRDBD_WT_, were characterized by worse scores than the EcRDBD_WT_ structure (see Table 2). However, all the results of the chemical denaturation experiments showed, that the application of computational methods for the EcRDBD redesign was an effective tool for finding substitutions that improved the domain's chemical stability. Two point mutants suggested by the program (M49W and L58F) turned out to have higher chemical stability than the EcRDBD_WT_.

Taking into account that RosettaDesign had been successfully used to design both chemically and thermally stable proteins [20], [22]–[24], we decided to verify the degree of thermal stability of the EcRDBD point mutants. The thermal denaturation measurements were supported by a CD spectroscopy. Due to the high content of secondary structures observable for all the analyzed domains, changes in ellipticity were measured at a wavelength of 222 nm (Figure 7). To determine the precise melting temperature (T_m_) value of each domain, the first derivatives of the CD-unfolding curves (dΘ/dT) were calculated using Jasco Spectra Analysis software (JASCO Corporation, Japan) (Figure 7, Insets and Table 2). First of all, the comparison of both unfolding curves of the EcRDBD_WT_ and UspDBD (see Figure 7K) showed a definitely lower stability in solution for the EcRDBD_WT_ with lower T_m_ value equal to 51.17°C (Table 2). The UspDBD is definitely more stable with its T_m_ value higher by 7°C than T_m_ of the EcRDBD_WT_. On the basis of our thermal denaturation results, the EcRDBD point mutants can be divided into three groups. The first group, consisting of the C61A and V64E point mutants, is described by a much lower T_m_ value than the EcRDBD_WT_. These results precisely confirmed the remarkable instability of the C61A and V64E point mutants (Figure 7D and E) which had been observed earlier during the chemical denaturation experiments (see Figure 6A). The CD-unfolding curves recorded for these two point mutants have a more linear than sigmoidal shape, with a full denatured state at 48°C and 56°C for the C61A and V64E mutants, respectively. None of the curves can be described as a two-state unfolding mechanism in which the folded protein is cooperatively converted to the unfolded form. Nevertheless, the derivative curves were calculated and both values of 31.57°C and 36.66°C were estimated for the respective (dΘ/dT) maxima as the apparent T_m_ (Table 2). The point mutants that belong to the second group are as follows: L16R, M49W and V64M. This group of mutants can be characterized by a moderate decrease of the T_m_ values in relation to the EcRDBD_WT_ (Figure 7, panels A, B and F, respectively). Notably, the M49W point mutant revealed a completely different stability profile in the presence of GdmCl than the L16R and V64M mutants (see Figure 6A, filled triangles, asterisks and x symbols for the M49W, L16R and V64M point mutants, respectively). Finally, the third group is represented by only one mutant, i.e. L58F. As shown in Figure 7C, the thermal stability of the EcRDBD was substantially increased by the L58F substitution and gave the T_m_ equal to 55.64°C (Table 2). Undoubtedly, with reference to the thermal and chemical denaturation experiments, this is the best substitution that considerably improves EcRDBD thermal stability. In conclusion, the L58F mutation caused both higher stability of the EcRDBD presented in the chemical (Figure 6A, open diamonds) and thermal (Figure 7C) denaturation experiments and higher affinity to the *hsp27_pal_* in the EMSA experiments (Figure 4A–B; lanes 5 and 15, bars 5 and 15). The substitution of the EcRDBD L58 residue was very interesting because of the Y residue at the corresponding sequence position in the UspDBD (see Figure 1A) and our results showed that this critical mutation makes the EcRDBD resemble the UspDBD.

### The design and analysis of multiple EcRDBD mutants

To investigate whether the combination of the individual point mutations in a single EcRDBD molecule could produce a synergistic effect beyond their individual contribution or not, the respective multiple mutants were generated as follows: L16R/L58F, M49W/L58F, L16R/M49W and L16R/M49W/L58F. On the basis of all the computational and experimental results, with particular emphasis on the chemical and thermal denaturation experiments, two substitutions at residues M49 and L58 were chosen as determining EcRDBD stability. Additionally, the analysis of the initial results of the EcRDBD mutants' design made by RosettaDesign, showed that the most significant domain stability improvement is achieved if the additive effects of served substitutions are summed up (data not shown). Therefore, it was decided to join the L16 residue to the M49 and L58 residue set selected for further study. We assumed that the L16R substitution might contribute to EcRDBD stability in conjunction with the M49W or/and L58F mutations. The multiple mutant structures were designed and evaluated using the RosettaDesign program [20], [21]. According to the results of the RosettaDesign scoring [21], [25], [26], the energetic preferential structures were in order of priority: L16R/M49W>L16R/M49W/L58F>L16R/L58F>M49W/L58F (see Table 2). The program unequivocally pointed at the great influence of the L16R substitution on EcRDBD stability, in contrast to the EcRDBD structures containing the L58F mutation, that had been signed with worse scores. These results are in agreement with early results of computational evaluation of the point mutants.

Site directed mutagenesis was used to obtain the three double and one triple mutant constructs. The proteins were overexpressed and purified to homogeneity (data not shown). The EMSA experiments were carried out to verify the multiple mutant *hsp27_pal_*-binding activities in the absence and presence of the UspDBD. The multiple mutants revealed a significant DNA-binding affinity as both the homo- and heterodimers (see Figure 4C–D). The experiments, performed with increasing amounts of either indicated homo- or heterodimers showed, in all cases, a full DNA saturation near 200 nM of protein concentration (data not shown). To compare the subtle differences between the *hsp27_pal_*-binding affinities of multiple mutants, further EMSA experiments were carried out for lower proteins' concentration, equal to 50 nM. None of the generated multiple mutants lost their affinity to the *hsp27_pal_* and UspDBD partners (Figure 4C–D). The M49W/L58F and L16R/M49W/L58F mutation sets, produced improvements in the homo- and heterodimer affinity to the *hsp27_pal_* (Figure 4C–D; lanes 3, 5, 10 and 12, bars 3, 5, 10 and 12) in comparison to the EcRDBD_WT_ (Figure 4C–D; lanes 6 and 13, bars 6 and 13). The remaining two double mutants, L16R/L58F and L16R/M49W, displayed similar DNA-binding characteristics as both the homo- and heterodimers (Figure 4C–D; lanes 2, 4, 9 and 11, bars 2, 4, 9 and 11) in comparison with the EcRDBD_WT_ (Figure 4C–D; lanes 6 and 13, bars 6 and 13).

The far-UV CD spectra of the EcRDBD multiple mutants were recorded in the same manner as described for the point mutants. The quantitative examination of all the CD spectra was performed by the CDPro package software [49] and the results were collected in Table 1. The comparison of the L16R/L58F, L16R/M49W and L16R/M49W/L58F CD spectra with the EcRDBD_WT_ spectrum revealed a high similarity between one another (compare panels G, I and J of Figure 5, respectively). The deconvolution of the CD spectra of the L16R/L58F, L16R/M49W and L16R/M49W/L58F mutants showed that the proportions of the individual secondary structures did not undergo substantial change (see Table 1). The CD spectra of the L16R, and L58F point mutants were significantly different from the EcRDBD_WT_ spectrum (see Figure 5A and C, respectively). However, some of the multiple mutants containing these two single mutations recover the spectrum characteristics of the EcRDBD_WT_ (compare panels A and C with panels G, I and J of Figure 5). The M49W/L58F mutant CD spectrum resembles the CD spectrum of the L58F point mutant (compare panels C and H of Figure 5). In the case of the M49W/L58F mutant, the F residue presence at position 58 exerted more influence on the DBD structure than the W residue at position 49 (compare panels B, C and H of Figure 5).

In conclusion, the M49W/L58F mutant was described by CD spectrometry as the only one multiple mutant which significantly differs with the EcRDBD_WT_. Our results suggest that the L58F substitution has a remarkable influence on EcRDBD secondary structure content. The CD spectra of the rest of the multiple mutants (L16R/L58F, L16R/M49W and L16R/M49W/L58F) are similar to the EcRDBD_WT_ CD spectrum. Moreover, the similarity between the L16R/M49W/L58F mutant and the EcRDBD_WT_ CD spectra indicates that the EcRDBD reveals a high degree of structural adaptability and plasticity.

### The stability evaluation of the EcRDBD multiple mutants

Next, we studied the multiple EcRDBD mutants' unfolding processes using GdmCl as a chemical denaturing agent. The denaturation profiles of the wild-type and mutated EcRDBDs are significantly different (see Figure 6B). The M49W/L58F construct turned out to be the most stable multiple mutant (Figure 6B, filled triangles) in comparison with the EcRDBD_WT_ (Figure 6B, filled circles). Interestingly, this double mutant showed a lower chemical stability than the M49W point mutant and simultaneously a higher stability than the L58F mutant (compare Figure 6B, filled triangles with Figure 6A, filled triangles and open diamonds). The concentrations of GdmCl corresponding to 50% unfolded M49W, L58F and M49W/L58F mutants were 1.43 M, 1.00 M and 1.31 M, respectively (see Table 2). The chemical denaturation profiles of the triple mutant (L16R/M49W/L58F) and EcRDBD_WT_ nearly overlapped with each other. They are very similar to each other at lower concentrations of GdmCl (up to 1 M) and, in both cases, full denaturation was achieved at nearly 2.5 M GdmCl. Both the L16R/M49W/L58F mutant and EcRDBD_WT_ were described by comparable values of the C_1/2_ parameter (0.73 M and 0.74 M, respectively). The L16R/L58F and L16R/M49W mutants demonstrated lower stability in comparison to the EcRDBD_WT_ with C_1/2_ of 0.44 M and 0.57 M, respectively. In conclusion, only one multiple mutant (M49W/L58F) showed a significantly higher chemical stability than the EcRDBD_WT_ which once again emphasizes the remarkable influence of the L58F substitution on EcRDBD structure and stability.

The thermal stability of the multiple mutants was monitored by the ellipticity signal at 222 nm which is predominantly associated with the secondary structure content (see panels G–J of Figure 7). The CD denaturation curves were analyzed in detail by calculation of their first derivatives (dΘ/dT) and the respective T_m_ values were collected in Table 2. Highly cooperative unfolding processes were obtained for all of the multiple mutants. In the case of the L16R/L58F and L16R/M49W mutants, the invariable α-helix content remained stable in the respective ranges between 16–31°C and 19–30°C (see panels G and I of Figure 7). This would indicate the presence of stable domain intermediates on the unfolding pathways [50]. A further heating of each of these double mutant probes, had the effect of gradual L16R/L58F unfolding with T_m_ 53.75°C, whereas the sharper bias of the L16R/M49W mutant curve toward an unfolded state gave T_m_ 44.13°C (panels G and I of Figure 7 and Table 2). Interestingly, the T_m_ of the L16R/M49W (44.13°C) is close to the T_m_ values determined for each of the L16R and M49W point mutants (45.11°C and 44.33°C, respectively). The thermal denaturation profiles of the M49W/L58F and L16R/M49W/L58F mutants could be interpreted as apparently cooperative two-state unfolding processes indicating the absence of any significant populations of intermediates (Figure 7, panels H and J). The M49W/L58F mutant turned out to be one of the most noteworthy domains because of its higher T_m_ value (54.22°C) in comparison with the EcRDBD_WT_ T_m_ (51.17°C). This double mutant was also described with the highest value of C_1/2_ (1.31 M, see Table 2) in chemical denaturation experiments (see Figure 6, filled triangles). The M49W/L58F and L16R/L58F mutants proved to have identical thermal unfolding curves in the temperature range between 40–70°C (Figure 7L). Probably, neither the L16R nor M49W but the L58F substitution determined this kind of unfolding pathway of both double mutants. On the basis of these results we can state that our early assumption concerning the L16R contribution to EcRDBD stability in conjunction with the M49W or/and L58F mutations was not entirely accurate. In conclusion, the most significant EcRDBD chemical and thermal stability improvement was achieved not by multiple mutations but through a single-point amino acid substitution at the position 58. Both the L58F and M49W/L58F mutants are characterized by higher C_1/2_ and T_m_ values than the EcRDBD_WT_ (Table 2) and specific binding to the *hsp27_pal_* as both the homo- and heterodimers (compare Figure 4A–B; lanes 5 and 15, bars 5 and 15 with Figure 4C–D; lanes 3 and 10, bars 3 and 10). Interestingly, there were no synergistic effects on the thermal and chemical stability of the EcRDBD, originating from the L16R, M49W and L58F substitution (Figure 8).

## Discussion

In our research we describe the molecular basis of the relatively low stability of the EcRDBD in comparison to the UspDBD [12]. The aim of our research was to map the *D. melanogaster* EcRDBD amino acid sequence positions which cause intriguing instability of the domain. We indicated five amino acid residues (L16, M49, L58, C61 and V64), the substitutions of which, modulate DBD stability. Despite the fact that all of the designed substitutions changed the EcRDBD structure (see Figures 1, 2 and 5A–F), none of them abolished EcRDBD ability to interact specifically with the UspDBD and *hsp27_pal_* (see Figure 4A–B). These observations concur with the great plasticity and structural adaptability of the EcRDBD described previously [12], [51]. One significant example of EcRDBD structure plasticity was the triple mutant L16R/M49W/L58F which seemed to have a similar structure to the EcRDBD_WT_, based mainly on their comparable secondary structure contents (see Figure 5J and Table 1). Interestingly, the L16R/M49W/L58F mutant turned out to have a higher affinity to the *hsp27_pal_* than the EcRDBD_WT_ (see Figure 4C–D; lanes 5, 6, 12 and 13, bars 5, 6, 12 and 13). The conformational stability of the L16R/M49W/L58F mutant is on the same level as EcRDBD_WT_ stability (Table 2 and Figure 8). The comparison of the L16R/M49W/L58F mutant with the L16R, M49W and L58F point mutants and M49W/L58F double mutant showed that the L16R substitution acts as if it keeps the DBD structure unchanged (compare Figure 5A–C with Figure 5G–I and Figure 5J). On the other hand, the L16R mutation decreased the stability of the domain (Table 2). The M49W and L58F substitutions had a great impact on both the DBD backbone conformation and stability. However, no synergistic effects were noticed (Table 2 and Figure 8).

The results of our chemical and thermal denaturation experiments turned out to be inconsistent with the RosettaDesign scoring (Table 2 and Figure 8). According to the computational analysis, whereas the V64E, V64M and C61A point mutants were distinguished with the best scores, they were experimentally proven to be the most unstable domains (see Table 2). The M49W, L58F and L16R point mutants were described by worse scores than the EcRDBD_WT_. In conclusion, the RosettaDesign program was helpful in the rational design of the EcRDBD mutants, without losing the ability to interact specifically with the UspDBD and *hsp27_pal_*. However, only a few residues proved to be meaningful for EcRDBD stability.

Interestingly, our *in silico* studies selected the set of amino acid residues almost exclusively in the region between M49 and V64 residues. These substitutions should lead to improvements in the conformational stability of the EcRDBD and because of this should be the key determinants of the low stability of the EcRDBD molecule. Additionally, one amino acid residue (L16) was located near the N-terminal end of the EcRDBD (see Figure 1A). The L16R substitution did not significantly change the general DBD fold (see Figure 1E and Figure 2A). The L16R mutant was characterized by a lower secondary structure content than the EcRDBD_WT_ (see Figure 5A and Table 1). However, this point mutant demonstrated a similar level of conformational stability and affinity to the response element as the wild-type domain (see Figure 4A–B, Table 2 and Figure 8). The rest of the analyzed substitutions (M49W, L58F, C61A, V64E and V64M) had considerable influence on both EcRDBD functionality and conformational stability (Table 2 and Figure 8). Two of them (M49W and L58F) contribute to EcRDBD stability which was clearly shown in our chemical and thermal denaturation experiments performed for both the point mutants and the M49W/L58F double mutant (Table 2 and Figure 8). In particular, EcRDBD chemical stability was increased by mutations of both the M49 and L58 residues, whereas thermal stability was increased only by a substitution at position 58. The L58 residue is crucial for a determination of both the adapted fold and stability of the EcRDBD. The L58F substitution led to a reduction of EcRDBD secondary structure content (see Table 1), but it also improved the chemical and thermal stability of the domain (Table 2 and Figure 8). Indication of L58 residue as a target point of EcRDBD instability was an interesting result of our *in silico* predictions. A favorable arrangement of both W49 and F58 aromatic residues inside the EcRDBD hydrophobic core additionally confirmed a high level of domain plasticity and adaptability. These results highlight the influence of aromatic side chains on both DBD stability improvement and plasticity exhibition in a general perspective [34], [37], [38], [51], [52]. Moreover, the positions 49 and 58 in the EcRDBD amino acid sequence can be taken as a reference point of structural analysis of other nuclear receptor DBDs. The alignment of EcRDBD amino acid sequences from *Drosophila* and other species showed that all the EcRDBDs have M49 and L58 residues. This result suggests that other EcRDBDs might exhibit the same instability as *D. melanogaster* EcRDBD. However, further analysis supported by *in silico* and *in vitro* studies is required to confirm this suggestion.

Our *in silico* and *in vitro* studies showed that the molecular basis of conformational stability of the EcRDBD was driven by EcRDBD-specific amino acid residues (M49 and L58) located within the α-helix 2 of the DBD. This α-helical fragment of the EcRDBD is built mainly by amino acid residues which are highly conserved within the nuclear receptor family [9], [11], [12], [30]. However, there are also a few amino acid residues with undetectable levels of conservation. Three of these non-conserved residues (M49, L58 and V64) were deliberately selected by the RosettaDesign program for substitution leading to EcRDBD stability improvement (see Figure 1A). These residues are strictly conserved in EcRDBDs (see Figure S1). The replacement of the M49 and L58 residues with aromatic side chains (W and F, respectively) prove to have a significant influence on the conformational stability of the domain. Interestingly, there are more residues in the EcRDBD amino acid sequence which are non-conserved within the nuclear receptor family and not involved in the UspDBD and *hsp27_pal_* binding [11], [12], but none of these were selected for the domain stability redesign. The MD simulation results also indicate that α-helix 2 represents a specific structural element of the EcRDBD which introduces structural instability. As shown in Figure 3, RMSF values were higher for the α-helix 2 than α-helix 1 of the EcRDBD_WT_. The average amplitude of side chain motions within these two regions of the EcRDBD structure (H1 and H2) was similar only for the L16R and L58F point mutant structures (see panels A and C of Figure 3, respectively). Moreover, the RMSF profile of the L58F point mutant structure turned out to be similar to the RMSF profile calculated for the UspDBD (see Figure 3H) − having Y residue at position 58. This result suggests that L58 in the EcRDBD amino acid sequence is the key residue which differentiates the hydrophobic cores of the *D. melanogaster* UspDBD and EcRDBD_WT_. Thus, the L58 residue is potentially the main reason for the completely divergent stability of the EcRDBD. This insight into EcRDBD instability caused by the non-conserved amino acid residues located within α-helix 2 of the domain provides many points of discussion, especially with reference to the different pathways of the chemical and thermal unfolding processes described for the EcRDBD mutants. Our results might serve as a benchmark for further studies of the intricate nature of the EcRDBD.

## Materials and Methods

### The computational design method

The RosettaDesign program [20] was used to obtain potentially stable EcRDBD mutants. The target mutant structures were designed using the Metropolis Monte Carlo procedure as described previously [21], [22]. The crystal structure of the EcRDBD_WT_ bound to the *hsp27_pal_* and UspDBD (PDP code: 2HAN) was used as a template. The *hsp27_pal_*, UspDBD, water molecules and one of the double conformations of the EcRDBD residues (R32, R57, Q60 and R73) were removed. The EcRDBD residues subject to redesign were limited to those contributing to neither the EcRDBD_WT_-UspDBD, EcRDBD_WT_-*hsp27_pal_* interactions nor coordinating the zinc ions [6], [11]. The backbone coordinates were held constant and the sequence space was searched by the Metropolis Monte Carlo sampling using the Dunbrack backbone-dependent rotamer library of the possible chi-1, chi-2 and chi-3 angles of rotation defined for side chain models [27]. Fifty independent runs were done in each round.

The RosettaDesign program was used to define the set of amino acid sequence positions that form the hydrophobic core of the EcRDBD_WT_. The energy calculations were performed for the EcRDBD_WT_ structure, assuming a fixed backbone and no residue substitutions. In the first round of the calculations, all the amino acid sequence positions were labeled with the native-amino-acid (NATAA) parameter. All the side chain conformers from Dunbrack's library [27] with the extra chi-1, chi-2 and chi-3 dihedral angles were considered. The second round of the calculations was carried out using the native-amino-acid-and-rotamer (NATRO) parameter for all the residues. The results obtained during both the rounds were compared with each other and analyzed. In this approach, an energy-based criterion was assumed as follows: if the sum of the Lennard-Jones attractive energy (E_atr_), Lennard-Jones repulsive energy (E_rep_) and the Lazaridis-Karplus solvation energy (E_sol_) terms of a given residue is less than or equal to −1.9, the residue will be classified as the hydrophobic core of the domain [53]. In the same manner, the calculations were performed for the UspDBD structure.

### The molecular dynamics simulations

All molecular modeling calculations, including molecular dynamics (MD) simulations and their analysis were carried out using the AMBER Molecular Dynamics Package [44]. The initial models were constructed in two ways: by extracting the EcRDBD_WT_ and UspDBD structures from their heterocomplex crystal data [11] and by removing hydrogen atoms from the RosettaDesign resultant structures of the EcRDBD point mutants. The addition of the missing hydrogen atoms was carried out using the *leap* program from the AMBER package, assuming a protein in a neutral pH environment. Each protein was centered in a cubic box and then solvated with water molecules. The dimensions of the simulation box were chosen to be large enough to include at least 0.8 nm of solvent on each side of the protein molecule. The counter ions (Cl^−^) were added to achieve a neutral simulation box [54]. The parm99 version of the all-atom AMBER force field was used for all model systems.

The energy minimization procedures and the MD simulations were carried out using the *sander* program from the AMBER package. The energy minimization procedure was carried out in several steps to allow the gradual relaxation of the system. Firstly, the *steepest descent* method was followed by the *conjugate gradient* minimization algorithm. The production of the MD was performed in a 10 ns time period (including 300 ps of equilibration) at a constant temperature of 300 K (ensemble NVT). All the simulations were performed with the periodic boundary condition at the desired temperature using an external bath with a constant time integration step set equal to 2 fs. No constraints were imposed during the simulations [44]. The same energy minimization and the MD procedures were applied to all analyzed systems. The data was collected every 1 ps. The time-averaged properties obtained from the resultant trajectories were further compared to the static values and described geometry of the energy minimized models. The atomic positional fluctuations for Cα atoms and the average mass-weight of each residue were calculated by the *ptraj* program and represented by the root-mean-square-deviation (RMSD) and root-mean-square-fluctuation (RMSF) parameters, respectively [44].

### The construction of expression vectors; site-directed mutagenesis, expression and purification of the DBDs

The plasmid pGEX-2T (Amersham Biosciences, Freiburg, Germany) was used for the expression of the DBDs in fusion with the *Schistosoma japonicum* glutathione-S-transferase (GST) in *Escherichia coli* strain BL21(DE3)pLysS (Novagen, Germany). The construction of the expression plasmids for the wild-type EcR and the Usp GST-DBD was described previously [55]. The PCR-based megaprimer method for the site directed mutagenesis [46] was used to generate the cDNAs coding the EcRDBD mutants. The plasmid template for the EcR GST-DBD mutants was constructed as described previously [12]. The expression and purification of the EcRDBD_WT_, UspDBD and EcRDBD mutants were performed according to the procedure described previously for the UspDBD with a deleted C-terminal sequence [5].

### Protein concentration

The concentrations of the purified proteins were determined spectrophotometrically at 280 nm. The web-based ProtParam software [56] was used to estimate the molar extinction coefficient of the proteins.

### DNA-binding assay

Electrophoretic mobility shift assay (EMSA) experiments were performed as described previously [12]. The quantitative analysis was carried out using a Fuji Film FLA-3000 Fluorescent Image Analyzer. A digital densitometric analysis of all images was performed using AIDA Bio-Package software (Raytest Isotopenmeβgeräte GmbH, Germany).

### Chemical denaturation

Protein denaturation profiles were constructed on the basis of fluorescence measurements, using a FLUOROLOG-3 fluorometer (Spex, Jobin Yvon Inc., France) and an Auto Titration Injector F-3006 (HORIBA Instruments Inc.). The excitation and emission wavelengths of λ_ex_ = 275 nm and λ_em_ = 303 nm, respectively, were used for the UspDBD, EcRDBD_WT_ and the following EcRDBD mutants: L16R, L58F, C61A, V64M, V64E and L16R/L58F. The M49W, M49W/L58F, L16R/M49W and L16R/M49W/L58F mutants were analyzed using the λ_ex_ = 282 nm and λ_em_ = 351 nm. All measurements were performed at 20°C. The proteins were in a phosphate buffer (50 mM Na_2_HPO_4_, 250 mM NaCl, 5 µM ZnCl_2_, 1 mM 2-mercaptoethanol, pH 7.8) at a concentration of 2.5 µM.

The fluorescence measurements were performed in several steps. In the first step, the protein sample was incubated at 20°C and fluorescence changes were measured at time intervals of one minute. After stabilization of the fluorescence values, the protein sample was titrated with a concentrated stock of guanidine hydrochloride (GdmCl) solution (7.0 M). To obtain the desired denaturant concentration, the defined volumes of the samples were withdrawn from the incubation mixture, and, subsequently, corresponding volumes of the GdmCl solution were added to the mixture to acquire a final volume of 500 µl. Next, the protein sample was stirred with a titrator syringe and it was incubated to equilibrate for 5 min. Then the fluorescence was measured, as described above.

Each protein titration was carried out using GdmCl in a concentration range from 0 to 5 M and all data points were normalized to fraction unfolded scale, considering changes in the protein and denaturant concentration.

### Circular dichroism spectra and thermal denaturation

The circular dichroism (CD) spectra were performed using a J-710 spectropolarimeter (Jasco Corporation, Japan) at 1 nm increments between 260 and 196 nm in a 0.1 cm pathlength cuvette. Three scans (speed 20 nm/min., response time 4 sec., sensitivity standard 100 mdeg) were averaged for each protein sample and baseline (50 mM Na_2_HPO_4_ buffer, pH 7.8, 250 mM NaCl, 5 µM ZnCl_2_, 1 mM 2-mercaptoethanol). All the CD experiments were repeated tree times. The ellipticity data was collected on the basis of the actual temperature inside the CD sample cell, determined from a thermocouple reading. The measurements were performed at 20°C. Each protein concentration was approximately 0.15 mg/ml. Using Jasco Spectra Analysis software (JASCO Corporation, Japan), the averaged baseline spectra were subtracted from the corresponding averaged protein spectra, smoothed with a binominal filter (with repeat time value equal to 1) and scaled to molar residue ellipticity units (Θ_MRE_ in degree×centimeter^2^×decimole^−1^). The secondary structure analyses were undertaken with CDPro package software [49] using SELCON3 [57], CDSSTR [58] and CONTIN/LL [59] algorithms with the reference to data set 7 (SDP48) [60]. The SDP48 data set was chosen in our study due to spectra of both folded and denatured proteins included in the training dataset. Therefore, set 7 was expected to be more appropriate as a reference database than those based on only folded proteins. Indeed, set 7 produced the most reasonable results from among all the available reference databases (data not shown). The RMSD parameter was calculated for each analysis. It is a measure of the fit quality of the calculated spectrum to the experimental data. Low values of RMSD suggest the calculated and experimental spectra are consistent.

Thermal denaturation was determined by measuring the ellipticity at 222 nm (Θ_222_) as a function of temperature between 4 and 90°C. Each probe was heated by 1°C/min. and the Θ_222_ was measured in time-intervals of 20 seconds. The protein sample preparation procedure was the same as described for the CD spectra experiments.

## Supporting Information

Figure S1 **Sequence comparison of DBDs from EcR and Usp with other nuclear receptor DBDs.** The residue numbering is relative to the first C residue coordinating the zinc ion of the DBD zinc module. Pink asterisks indicate the zinc-coordinating cysteines, and blue asterisks are the residues that form the hydrophobic core that stabilizes the domain. The *D. melanogaster* EcRDBD and UspDBD sequences are in yellow. The DBD sequence positions corresponding to the analyzed EcRDBD residues (L16, M49, L58, C61 and V64) were highlighted in gray, and the R16, F58 and M64 residues found at the aligned sequences were highlighted in pink.
(TIF)

Supporting Information S1 **Supplemental files (.pdb files) contained in the compressed directory file S1 include structures of the EcRDBD_WT_, its mutants and the UspDBD.** All.pdb files can be visualized with Open Pymol. The optimized structures of the EcRDBD_WT_, its mutants and the UspDBD are named respectively: EcRDBD_WT.pdb, EcRDBD_[mutant].pdb and UspDBD.pdb. The DBD structures obtained after 10 ns of MD simulation are named respectively: EcRDBD_WT_10ns.pdb, EcRDBD_[mutant]_10ns.pdb and UspDBD_10ns.pdb.
(ZIP)
