# Supplementary figures and images for: The Molecular Basis of Conformational Instability of the Ecdysone Receptor DNA Binding Domain Studied by In Silico and In Vitro Experiments
Source: PLoS One. 2014 Jan 23;9(1):e86052. doi: 10.1371/journal.pone.0086052 (PMC3900457; doi:10.1371/journal.pone.0086052)

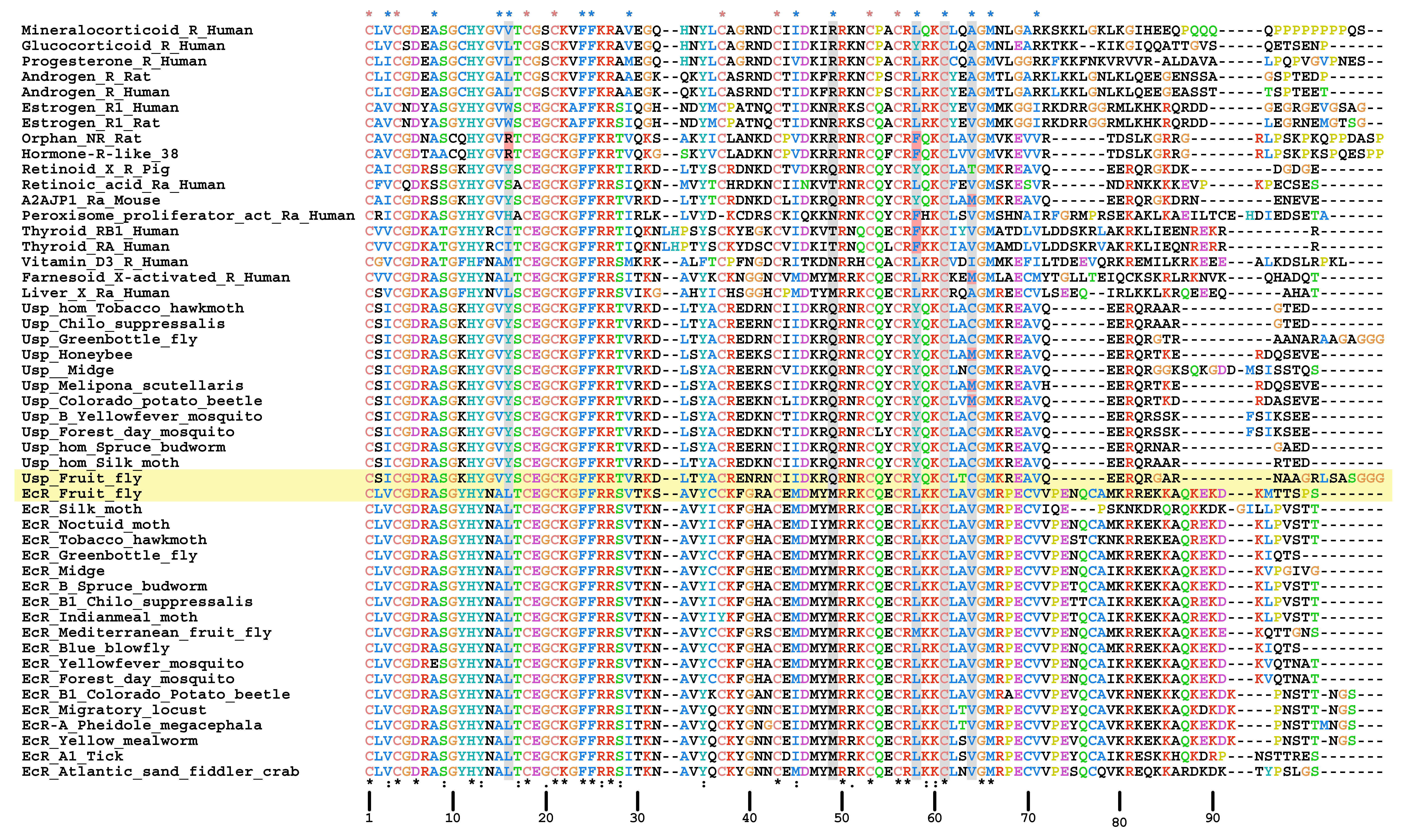

Supplement: Figure S1 — Sequence comparison of DBDs from EcR and Usp with other nuclear receptor DBDs. The residue numbering is relative to the first C residue coordinating the zinc ion of the DBD zinc module. Pink asterisks indicate the zinc-coordinating cysteines, and blue asterisks are the residues that form the hydrophobic core that stabilizes the domain. The D. melanogaster EcRDBD and UspDBD sequences are in yellow. The DBD sequence positions corresponding to the analyzed EcRDBD residues (L16, M49, L58, C61 and V64) were highlighted in gray, and the R16, F58 and M64 residues found at the aligned sequences were highlighted in pink. (TIF) [file pone.0086052.s001.tif]
